# Impact of clinical experience on type V pit pattern analysis using magnifying chromoendoscopy in early colorectal cancer: a cross-sectional interpretation test

**DOI:** 10.1186/1471-230X-14-100

**Published:** 2014-05-30

**Authors:** Taku Sakamoto, Takahisa Matsuda, Takeshi Nakajima, Yutaka Saito, Takahiro Fujii

**Affiliations:** 1 Endoscopy Division, National Cancer Center Hospital, 5-1-1 Tsukiji Chuo-ku, Tokyo 104-0045, Japan; 2 TF clinic, 4-13-11 Ginza Chuo-ku, Tokyo 104-0045, Japan

**Keywords:** Magnifying chromoendoscopy, Early colorectal cancer, Pit pattern, Type V pit

## Abstract

**Background:**

Although type V pit pattern analysis is effective in determining the invasion depth of early colorectal cancers, the clinical results may vary because findings are operator-dependent. This study aimed to assess the benefits of type V pit pattern analysis in estimating the invasion depth using magnifying chromoendoscopy compared to that with conventional colonoscopy.

**Methods:**

A cross-sectional interpretation test involving 32 endoscopists with varying levels of experience performing colonoscopies was conducted. Fifty histopathologically diagnosed cases of intramucosal or submucosal cancer were selected retrospectively. The lesions were classified as superficial or deep by the endoscopists, based on magnifying chromoendoscopic and non-magnifying endoscopic images. The endoscopists were classified into 3 groups based on the number of colonoscopies performed: I (<500), II (501–5000), and III (>5000). Differences in the interpretation of invasion depth between group III and groups I and II were assessed using the Mann–Whitney U test.

**Results:**

There was no significant difference in the median number of correct interpretations using non-magnifying endoscopic images among the groups. However, a significant difference (P = 0.007) was observed between the results of groups III and I when the analysis was performed using magnifying chromoendoscopic images.

**Conclusions:**

When performed by less experienced endoscopists, pit pattern analysis of colonic lesions using magnifying chromoendoscopy is not a reliable modality for estimating invasion depth in early colorectal cancer.

## Background

Magnifying chromoendoscopy has been widely demonstrated to be effective not only in differentiating between colorectal neoplastic and non-neoplastic lesions but also in accurately diagnosing the invasion depth of early colorectal cancers [1-11]. Estimating the invasion depth of early colorectal cancer is considered crucial for making decisions about appropriate treatment protocols. There have been reports of significantly increased risk factors for lymph node metastasis of early colorectal cancers in cases where the lesions invaded the deep submucosa (SM-d, distance from the muscularis mucosae ≥1,000 μm). On the other hand, the risk of metastasis is low in the absence of lymphovascular invasion, poorly differentiated adenocarcinoma component, and budding finding [12-14]. Therefore, estimating the intramucosal or superficial submucosal (SM-s, distance from the muscularis mucosae <1000 μm) invasion is important to determine appropriate treatment protocols.

Proper interpretation of the type V pit pattern provides critical information for appropriate treatment of early colorectal cancer. Type V pit patterns exhibit mild irregularity (V_I_ mild), severe irregularity (V_I_ severe), and non-structured (V_N_) patterns; in Japan, lesions showing V_I_ severe and V_N_ pit patterns are associated with a high risk of deep submucosal invasion [15,16]. Previous studies have demonstrated good reproducibility in the analyses of colonic pit patterns [17,18]. However, estimating invasion depth using magnifying endoscopy is considered more operator-dependent than differentiating between neoplastic and non-neoplastic lesions. Furthermore, some variability may exist among endoscopists in the interpretation of the subcategories of type V pit patterns. Therefore, despite the demonstrated effectiveness of this technique, pit pattern analysis with magnifying endoscopy has not yet been widely accepted for the assessment of early colorectal cancers—especially in Western countries.

The primary aim of this study was to determine the added benefit of magnifying chromoendoscopy to the diagnostic accuracy of conventional colonoscopy in estimating the depth of invasion of colorectal neoplasms, using indigo carmine spraying. In addition, we attempted to assess possible differences between experienced and less experienced endoscopists in the assessment of the invasion depth of the lesions, and subcategories of type V pit patterns.

## Methods

### Definition of type V pit patterns

**Figure 1:**
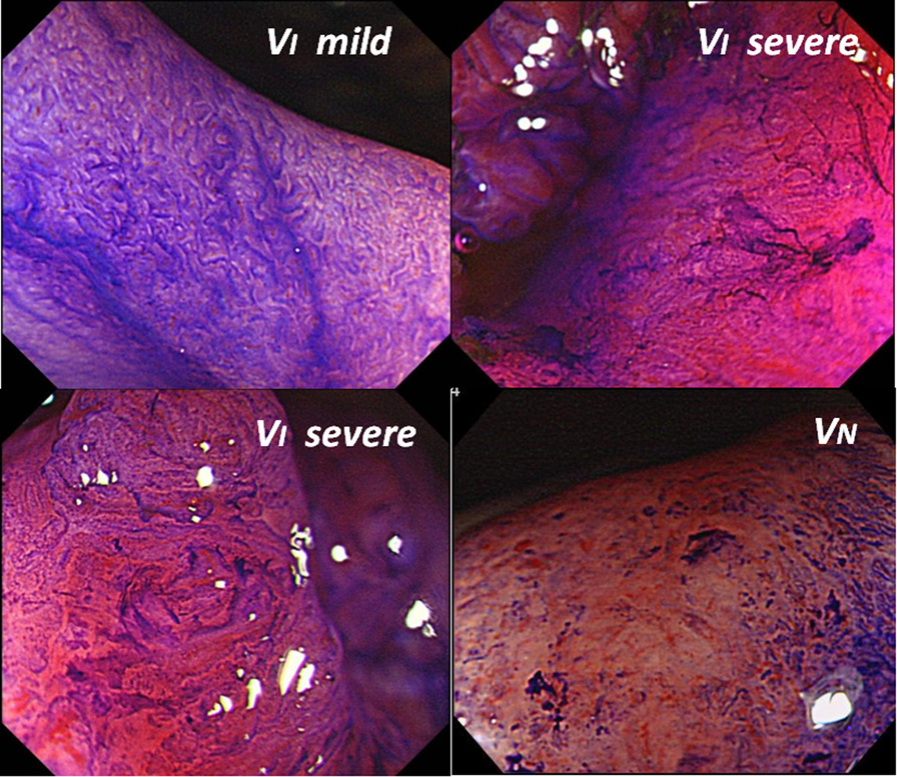
**Type V pit patterns consist of areas with irregular pits (type V**_**I**_**) and non-structured areas (type V**_**N**_**).** Type V_I_ severe pit patterns consist of areas with destroyed and badly damaged pits, including pits with irregular margins, narrowing, poorly demarcated boundaries, faded or unstained stromal areas, and signs of scratching.

According to the classification of colonic crypts described by Kudo and Tsuruta, type V pit patterns include areas with irregular crypts (type V_I_) and areas of apparent non-structure (type V_N_). Type V_I_ pit patterns are further subdivided into areas of mild irregularity (type V_I_ mild) and areas of severe irregularity, which exhibit destroyed and badly damage pits (type V_I_ severe). Tobaru et al. [15] defined type V_I_ severe pit patterns as areas with poorly demarcated pits and with pits showing faded or unstained stromal areas (Figure 1).

### Selection of cases

In a retrospective review of the colonoscopy examination database at the National Cancer Center Hospital (December 2008 to November 2009), we selected cases of colorectal cancer on the basis of the following criteria: 1) demonstration of histologically precise diagnoses; 2) detection by magnifying chromoendoscopy using crystal violet staining; 3) endoscopically diagnosed cases of early colorectal cancer that underwent subsequent endoscopic or surgical resection; and 4) cases with good quality images, as judged by an experienced endoscopist familiar with the histopathological diagnosis of colorectal cancers. The final histopathological diagnoses of invasion depth were 30 superficial (mucosa/submucosa, <1,000 μm) and 20 deep (submucosa, ≥1,000 μm) lesions. Other clinicopathological features of the selected cases are presented in Table 1.

**Table 1:** Clinicopathological features of the lesions selected for the study

**Macroscopic type**	**No. (%)**
0-Is	7 (14)
0-IIa, Is + IIa	19 (38)
LST-G-MIX	9 (18)
LST-NG-F	10 (20)
0-IIa + IIc, IIc + IIa, IIc	24 (48)
LST-NG-PD	24 (48)
Size of lesions (mean ± SD, mm)	27 ± 15
Invasion depth	No. (%)
Intramucosa/submucosa, <1000 μm	30 (60)
Submucosa, ≥1000 μm	20 (40)
Histology	No. (%)
Well diff. adenocarcinoma (W/D)	24 (48)
Low-grade atypia	21 (42)
High-grade atypia	4 (8)
Moderately diff. adenocarcinoma (M/D)	1 (2)

LST-G-MIX: laterally spreading tumors, granular, nodular mixed type; LST-NG-F: laterally spreading tumor, non-granular, flat elevated type; LST-NG-PD: laterally spreading tumor, non-granular, pseudo-depressed type.

### Selection of participants

Thirty-two Japanese endoscopists with various levels of experience at medical centers other than the National Cancer Center Hospital (where magnifying chromoendoscopy is utilized routinely for colonoscopies) participated in the study. All endoscopists were blinded to the clinical details of each case, outcome, including histopathological data and prescribed treatments. Participants were informed about the aims of the study at the outset, and that the study included 50 cases of colorectal neoplasms with no detailed information about invasion depth.

Each participant performed 2 independent assessments of endoscopic image data for each case, using images showing the relevant area for estimating invasion depth. The first session consisted of evaluating 2–4 endoscopic still images taken by conventional colonoscopy alone, and the second session consisted of evaluating a single conventional still image showing the entire lesion supplemented by 4 additional magnifying chromoendoscopy images; the images in the second assessment were shuffled to minimize the possibility of identifying or recognizing the lesion observed in the first assessment. The endoscopists classified the lesions as superficial or deep in each session, and classified the type V pit pattern by using chromoendoscopic images in the second session. The evaluation time in each session was limited to 2 min per case.

### Data analysis

We categorized the endoscopists’ level of experience into 3 groups depending on the number of colonoscopies each had performed: group I, <500 (12 endoscopists); group II, 501–5000 (10 endoscopists); and group III, >5000 (10 endoscopists). A Mann–Whitney *U* test was performed to assess the significance of differences between the depth invasion assessments of the 3 groups. We compared data from group I with that from groups II and III because the endoscopists assigned to Group III were highly experienced with colonoscopy and magnifying chromoendoscopy. Hence, Group III should be set as the reference for diagnostic ability. The kappa statistic was used to compare agreements for type V pit pattern classifications within groups.

### Ethics

The subjects were identified by reviewing the endoscopic database at our division. The study was conducted in accordance with the guidelines of our institutional review board, and was approved without the need for patients’ informed consent. All patients had provided written informed consent for the colonoscopy and endoscopic treatment.

## Results and discussion

**Figure 2:**
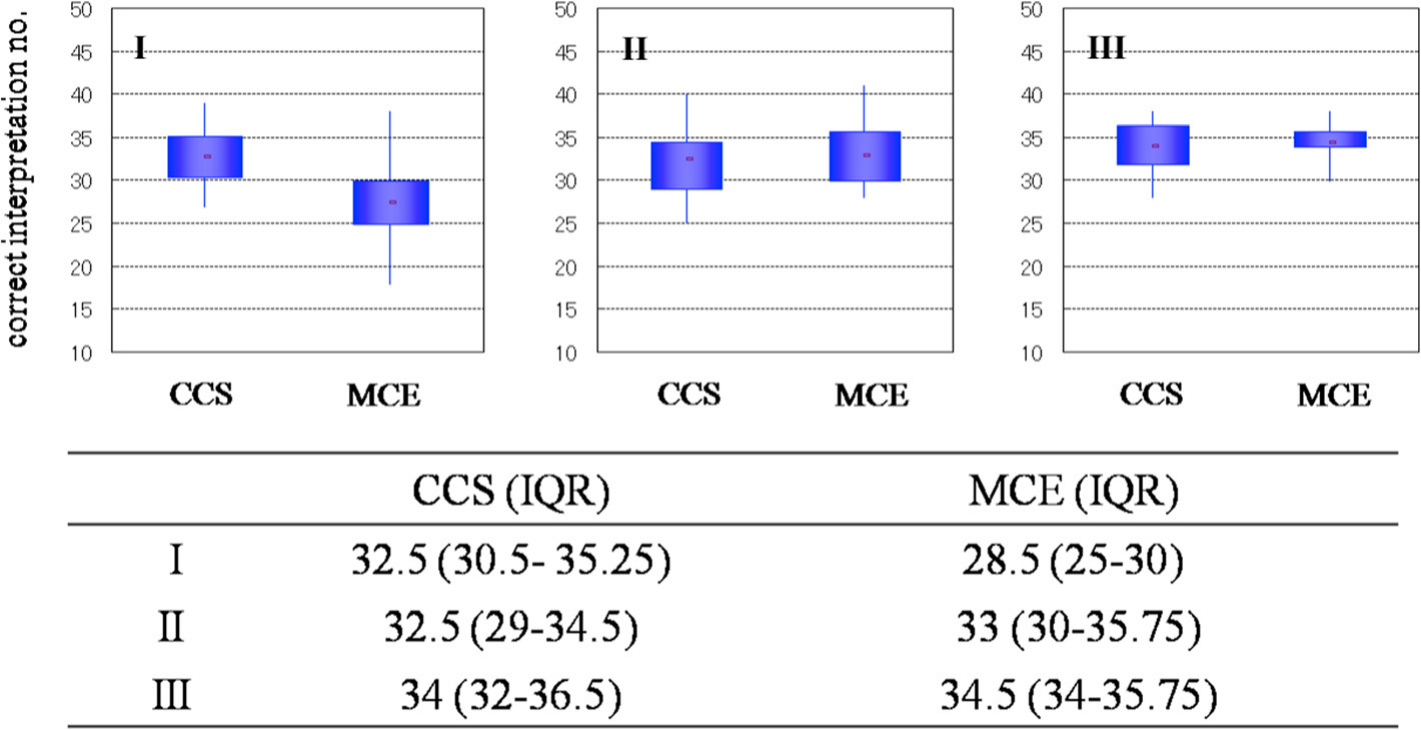
Differences in the number of correct interpretations of invasion depth by the different endoscopist groups (I, II, and III) for conventional colonoscopy (CCS) and magnifying chromoendoscopy (MCE).

The results of the endoscopist assessments are presented in Figure 2. For the first session (conventional colonoscopy, CCS), the number of correct interpretations of the invasion depth (median and interquartile ratio, IQR) were 32.5 (30.5–35.5) in group I, 32.5 (29–34.5) in group II, and 34 (32–36.5) in group III; no significant differences were noted between group III and the other groups. For the second session (magnifying chromoendoscopy, MCE), the number of correct interpretations was 28.5 (25–30) in group I, 33 (30–36) in group II, and 34.5 (34–36) in group III; significant differences were observed between groups III and I (P = 0.007). In both sessions, the smallest range of the number of correctly interpreted cases was observed in group III, which had the most experienced endoscopists. The kappa value for inter-observer agreement of type V_I_ sub-classification was 0.18 in group I, 0.18 in group II, and 0.48 in group III (Table 2).

**Table 2:** Inter-observer agreements for pit pattern classification within the 3 endoscopist groups

	**Kappa value**	**Agreement evaluation**
I	0.18	Slight
II	0.18	Slight
III	0.48	Moderate

Table 3 shows the clinicopathological features of the cases with less than half the number of correct diagnoses, as determined by the experienced colonoscopists in group III. There were 4 superficial and 2 deep cancers. Regarding the histopathological findings, all cancers were well-differentiated adenocarcinoma, with 3 of the 4 superficial lesions showing high-grade atypia and one showing low-grade atypia, and both deep lesions showing low-grade atypia.

**Table 3:** Difficult interpretations of cases by group III endoscopists using magnifying chromoendoscopy

**No. of correct interpretations**	**Size (mm)**	**Macroscopic type**	**Depth (μm)**	**Histology**	**Grade of atypia**
1	30	IIc	M	W/D	Low
1	25	IIa + IIc	M	W/D	High
1	20	IIa	M	W/D	High
1	15	IIa + IIc	SM-s (300)	W/D	High
1	45	IIa	SM-d (1300)	W/D	Low
2	18	Isp	SM-d (1700)	W/D	Low

M: intramucosa; SM-s: submucosa, <1000 μm; SM-d: submucosa, ≥1000 μm; W/D: well-differentiated adenocarcinoma.

To the best of our knowledge, this is the first report to compare the diagnostic ability of depth invasion in early colorectal cancers, using pit pattern analysis with magnifying chromoendoscopy, among endoscopists with varying levels of experience. In this study, the number of correct interpretations of invasion depth was lower in the second session test than in the first session test, in the inexperienced group. However, the number of correct interpretations tended to increase with experience, indicating that accurate estimation of invasion depth for early colorectal cancer using magnifying chromoendoscopy requires experience.

Regarding the efficacy of pit pattern analyses, it is important to differentiate between “differentiating neoplastic and non-neoplastic lesions” and “estimating depth invasion for neoplastic lesions” because the former is probably easier than the latter. Pit pattern analyses using magnifying chromoendoscopy enable us to accurately observe the surface histology of the lesions; some pit patterns show various degrees of type V irregularity related to the grade of atypia [1,19-26]. This is considered one factor that makes it difficult to accurately interpret type V pit patterns for inexperienced colonoscopists, and one reason magnifying chromoendoscopy has not became more widespread, despite its obvious diagnostic efficacy.

Regarding inter-observer agreements, our results showed a slight agreement in groups I and II and moderate agreement in group III. This finding indicates that magnifying chromoendoscopy for estimating the depth of early colorectal cancer might be clinically unreliable for inexperienced colonoscopists. However, the clinical value of magnifying chromoendoscopy is not only in the estimation of invasion depth, but also in the differentiation between neoplastic and non-neoplastic lesions. Typical adenomas usually show relatively regular tubular and/or villous structures histologically, and pit patterns in these lesions are less variable. Therefore, inexperienced endoscopists can easily differentiate between neoplastic and non-neoplastic lesions using magnifying chromoendoscopy.

Regarding the standardization of diagnoses by magnifying chromoendoscopy, the classification should be linked directly with the selection of appropriate treatments. The invasion depth of early colorectal cancer is usually judged from the accumulated data of serial observations, including conventional imaging without magnification. From this perspective, Matsuda et al. [6] reported the clinical classification “Invasive/Non-invasive pattern,” which includes conventional observations of lesion configuration, such as depression, large nodules, or reddened area. For differentiating between M/SM-s and SM-d lesions, interpretation using this invasive pattern showed a sensitivity of 85.6% and a specificity of 99.4% [6]. In this report, diagnostic accuracy sufficiently demonstrated the efficacy of magnifying chromoendoscopy, and the clear advantage of this classification was directly reflected in the choice of treatment: endoscopic or surgical resection. On the basis of the pit pattern classification, the invasive pattern might include some cases classified as V_I_ severe and V_N_ pit patterns. Therefore, it might be difficult to discriminate endoscopically between M/SM-s and SM-d cancers on the basis of magnifying chromoendoscopy results alone.

For experienced colonoscopists, magnifying chromoendoscopy is considered a complementary tool heightening the levels of diagnostic confidence and consensus, and contributing to high diagnostic accuracy. However, we should recognize the exceptional cases in which depth invasion is difficult to diagnose using magnifying chromoendoscopy. In this study, in some cases, the number of correct diagnoses was less than half the number, as determined by the experienced group III. It is generally considered that intramucosal tumors show histologic evidence of low-grade atypia, whereas the majority of infiltrative tumors exhibit high-grade atypia. Therefore, as discussed above, lesions showing highly irregular pits are considered as cases at risk for submcuosal infiltrating cancer. The possibilities of misdiagnosis on the basis of only pit pattern analysis are heightened in cases of invasive cancers with low-grade atypia or intramucosal cancers with high-grade atypia. When there is a discrepancy between the estimations of invasion depth using conventional colonoscopy and magnifying chromoendoscopy, other modalities such as “endoscopic ultrasonography,” “non-lifting sign,” or “narrow-band imaging” should be used in the evaluation.

There are some limitations inherent to our study. First, all evaluations were based on retrospective analyses of still images, which can differ considerably from in vivo endoscopic assessments of specific areas because the retrospective analyses exclude the possibility of direct examinations. Second, the small sample size may have introduced the possibility of beta error in the statistical interpretations of the diagnostic accuracy of magnifying chromoendoscopy in estimating invasion depth; in this study, we only evaluated the difference in diagnostic ability on the basis of colonoscopic experience. Therefore, this study may not be relevant for the evaluation of diagnostic performance of magnifying chromoendoscopy in estimating invasion depth.

## Conclusions

In conclusion, the results of the present study suggest that pit pattern analysis of colonic lesions with magnifying chromoendoscopy is not a reliable modality in estimating the depth of invasion by less experienced endoscopists. Thus, such diagnoses might improve with the level of experience of the endoscopist.

## Abbreviations

MCE: Magnifying chromoendoscopy; CCS: Conventional colonoscopy.

## Competing interests

The authors declare that they have no competing interests.

## Authors’ contributions

Conception and design: TS. Acquisition of data: TS. Analysis and interpretation of data: TS. Manuscript writing: TS, TM, TN, YS, and TF. Critical revision of manuscript: TM, TN, YS, and TF. Final approval of manuscript: TS, TM, TN, YS, and TF. All authors read and approved the final manuscript.

## Pre-publication history

The pre-publication history for this paper can be accessed here:

http://www.biomedcentral.com/1471-230X/14/100/prepub
